# Supplementary material for: Carbon Fiber-Reinforced Thermoplastic Composite Coatings for Steel Pipelines
Source: Polymers (Basel). 2024 Dec 5;16(23):3417. doi: 10.3390/polym16233417 (PMC11644091; doi:10.3390/polym16233417)
Supplement: Supplementary file 1 [file polymers-16-03417-s001.zip › polymers-3305126-supplementary.pdf]

Supplementary Information

Carbon Fiber -Reinforced Thermoplastic Composite Coatings for Steel Pipelines

Ahmed I. A. Abd El-Mageed [1,2,*], Mohamed M. Desouky [1], Mamdouh El-Sayed [1], Tarek Salem [3], Ahmed Bahgat Radwan [4], Mohammad K. Hassan [4], Affaf K. Al-Oufy [5,6] and H. M. El-Dessouky [1,7,*]

[1]Advanced Composites Research Lab, Faculty of Science, Galala University, Galala City 43511, Egypt.

[2]Colloids & Advanced Materials Group, Chemistry Department, Faculty of Science, Minia University, Minia 61519, Egypt.

[3]Dyeing, Printing and Auxiliaries Department, Textile Research Institute, National Research Centre, Cairo 12622, Egypt

[4]Center for Advanced Materials, Qatar University, Doha 2713, Qatar.

[5]Mechanical Engineering Department, Faculty of Engineering, Galala University, Galala City 43511, Egypt.

[6]Textile Engineering Department, Faculty of Engineering, Alexandria University, Alexandria 21544, Egypt.

[7]Physics Department, Faculty of Science, Mansoura University, Mansoura 35516, Egypt

[*]Corresponding Authors: Prof. Hassan El-Dessouky, h.el-dessouky@gu.edu.eg
Dr. Ahmed I. A. Abd El-Mageed, ahmed.abdelmageed@gu.edu.eg

Supplementary Table(s)

Table S1. Properties of Araldite®2011 adhesive as received.

Property	2011/A	2011/B	2011 (mixed)
Color - visual	Neutral	Pale yellow	Pale yellow
Specific gravity	1.15	0.95	1.05
Viscosity at 25 °C (Pas)	30-50	20-35	30-45
Pot life (100 gm at 25 °C)	-	-	100 minutes
Lab shear strength at 23 °C	-	-	> 19 MPa

Table S2. Fitting results obtained from the EIS measurements of the as-prepared coatings in 3.5 wt.% NaCl.

Sample	Rc k Ω cm^2	n_1	CPE1, μ s^n Ohm^{-1} cm^{-2}	Cc, μF cm^{-2}	Rct, k Ω cm^2	n_2	CPE2, μ s^n Ohm^{-1} cm^{-2}	Cdl, nF cm^{-2}	W μ Ω $cm^2s^{-1/2}$
**UD**	38	0.656	32	34	48	0.752	26	63	--
**Biaxial**	356	0.788	4.6	5.2	445	0.815	0.02	7	--
**Off-axis**	190	0.678	16	25	226	0.787	0.1	36	18

Supplementary Figure(s)

Materials and Methods

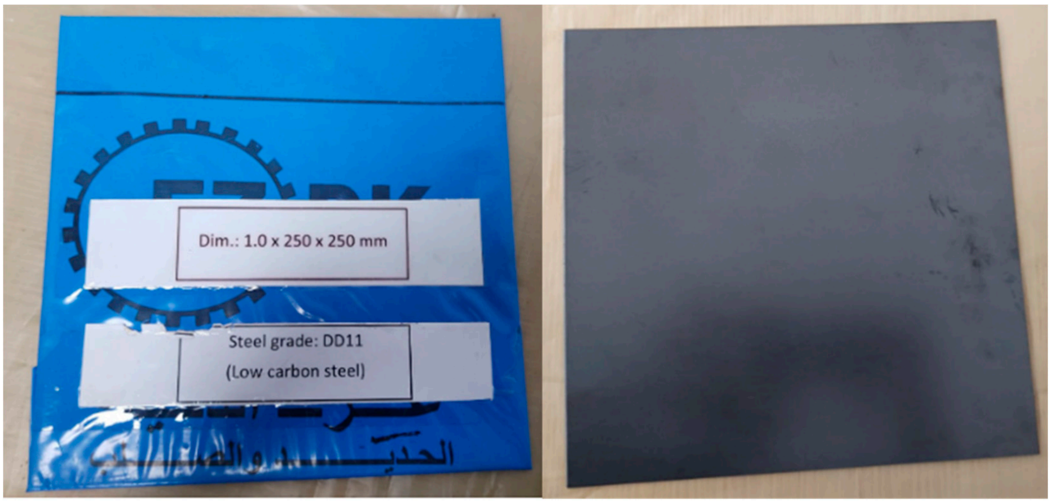

Figure S1. Low carbon-steel plates

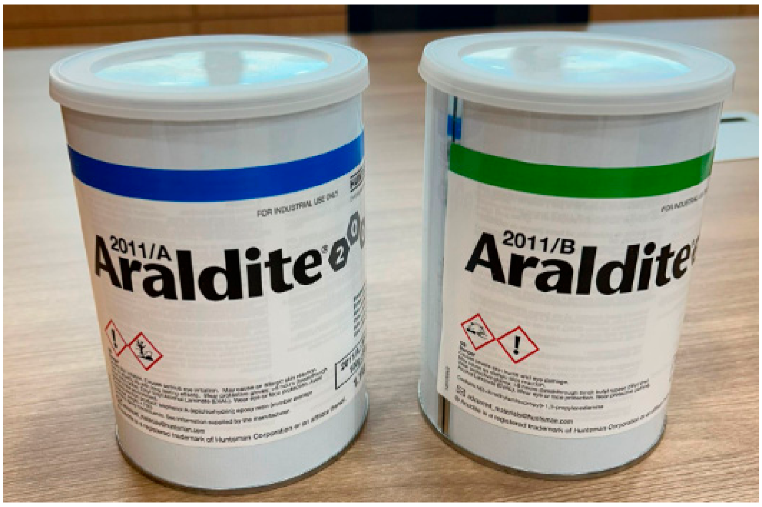

Figure S2. Araldite adhesive: Araldite® 2011/A & Araldite® 2011/B

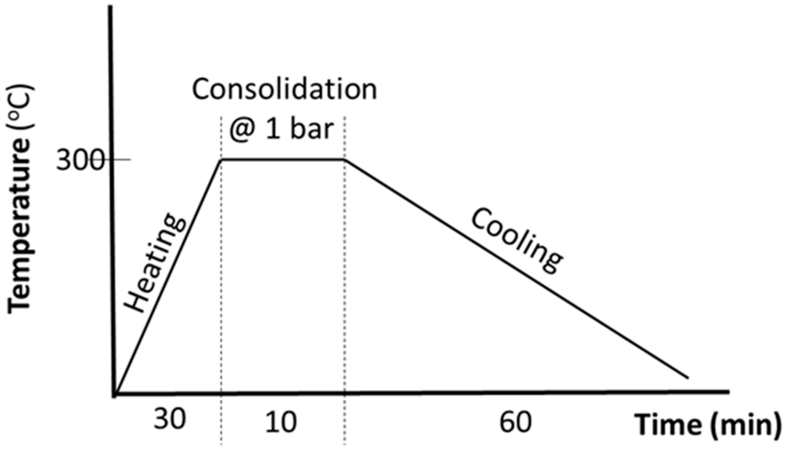

Figure S3. Consolidation profile used for manufacturing the CF/PPS composites

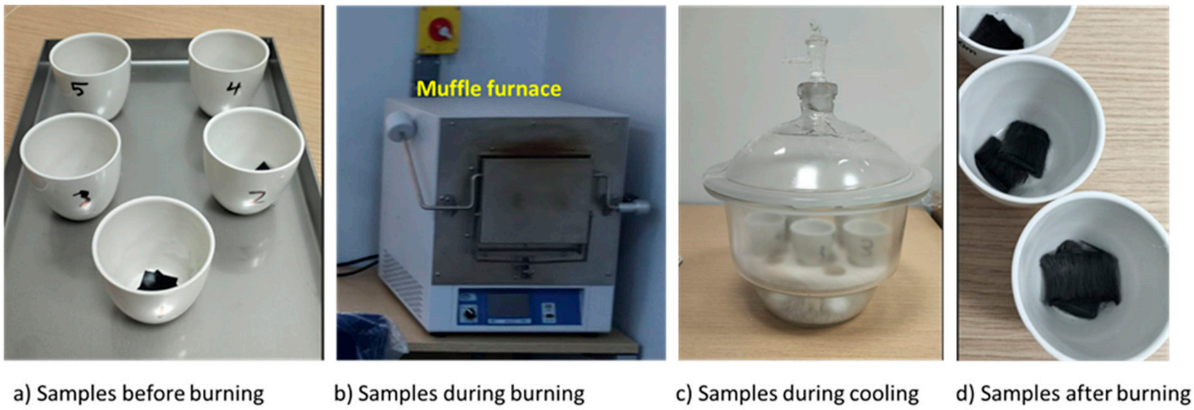

Figure S4. Burn-off process for composite specimens.

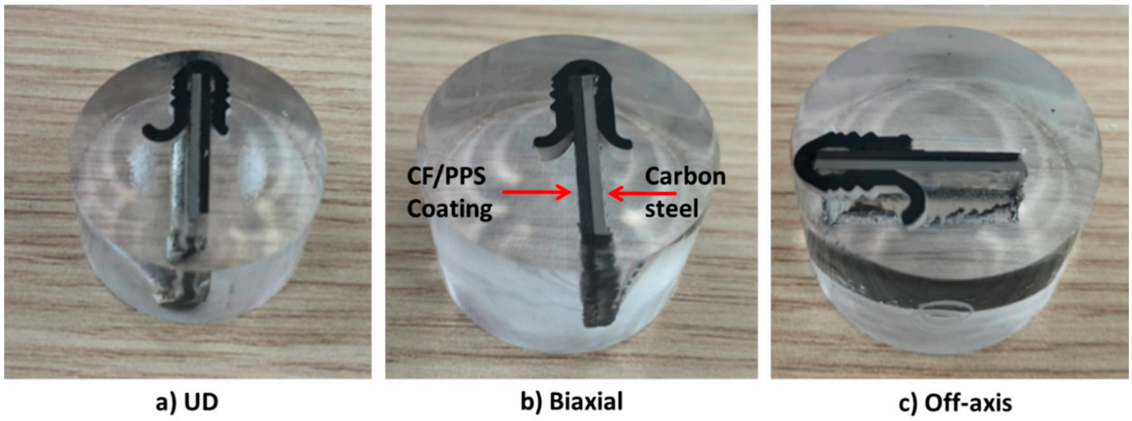

Figure S5. Microsections for optical microscopy.

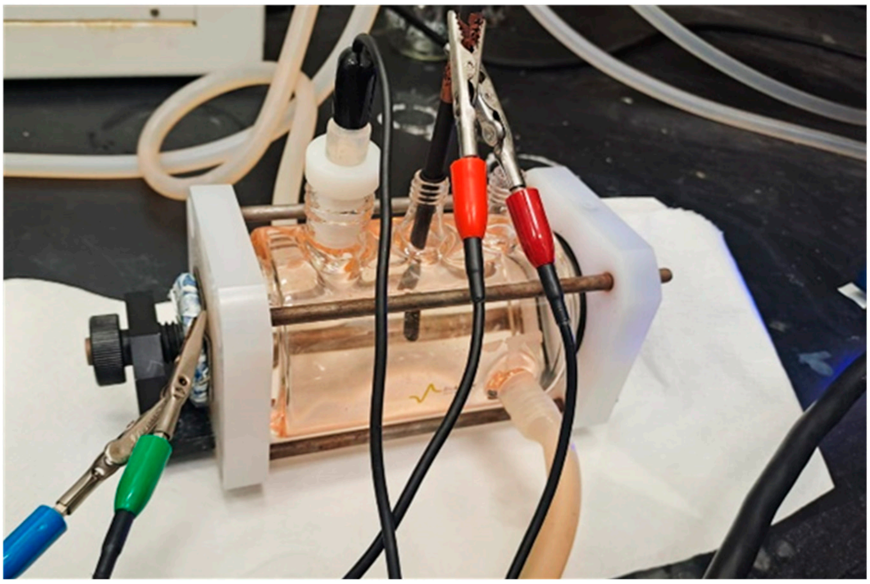

Figure S6. Setup for Electrochemical Impedance Spectroscopy (EIS) used in corrosion measurement experiments

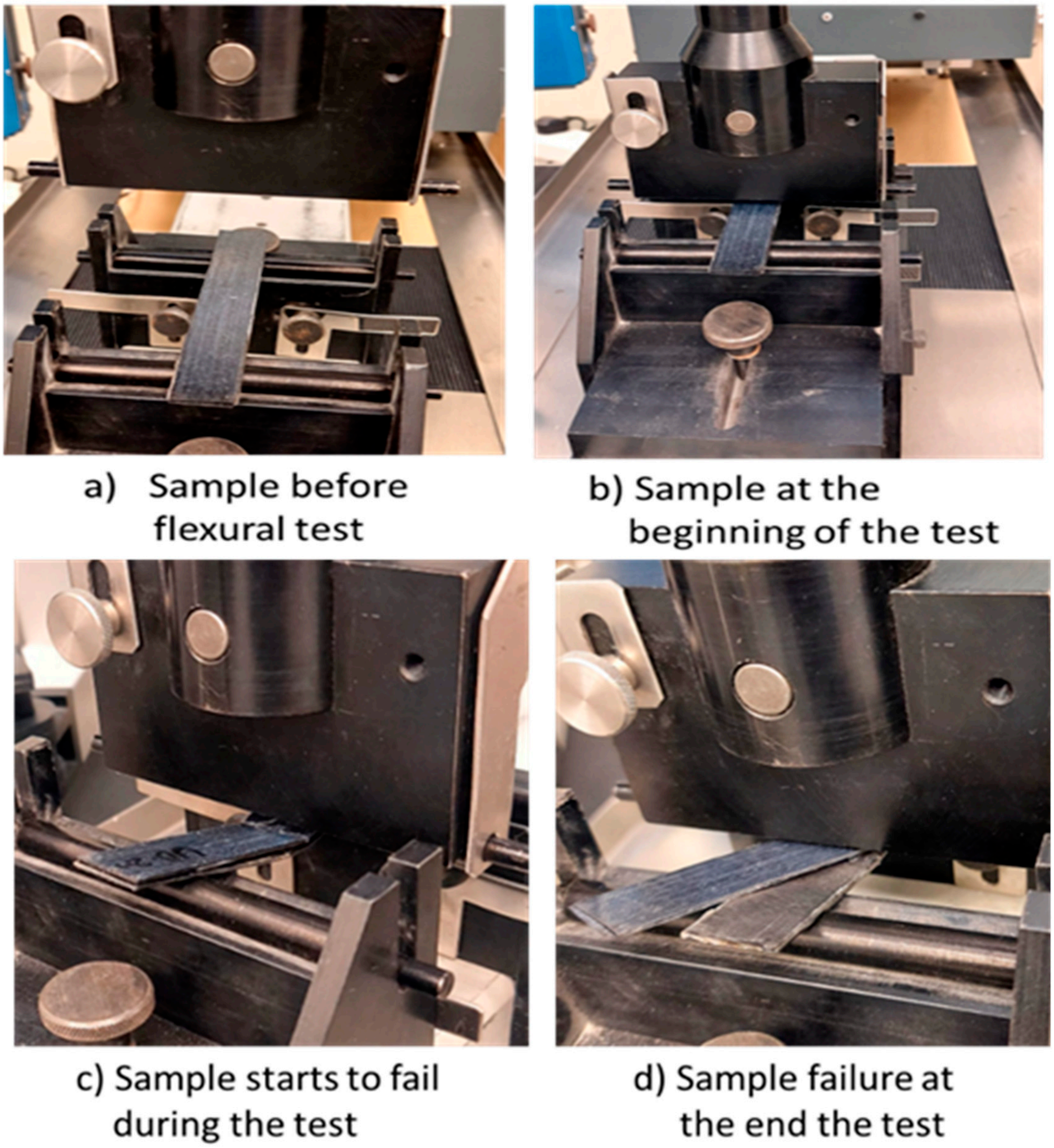

Figure S7. Set up of 3-piont bending test includes steps of flexural failure of test sample.

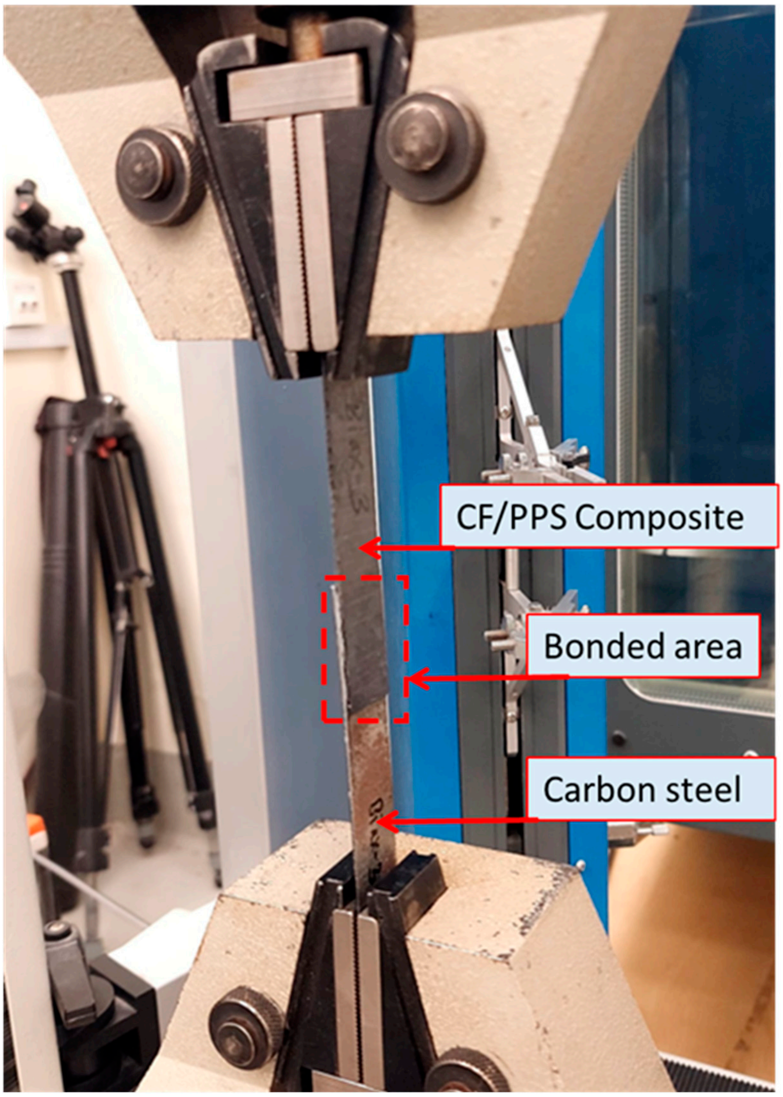

Figure S8. Set up of Single-Lap shear test.

Results and Discussion

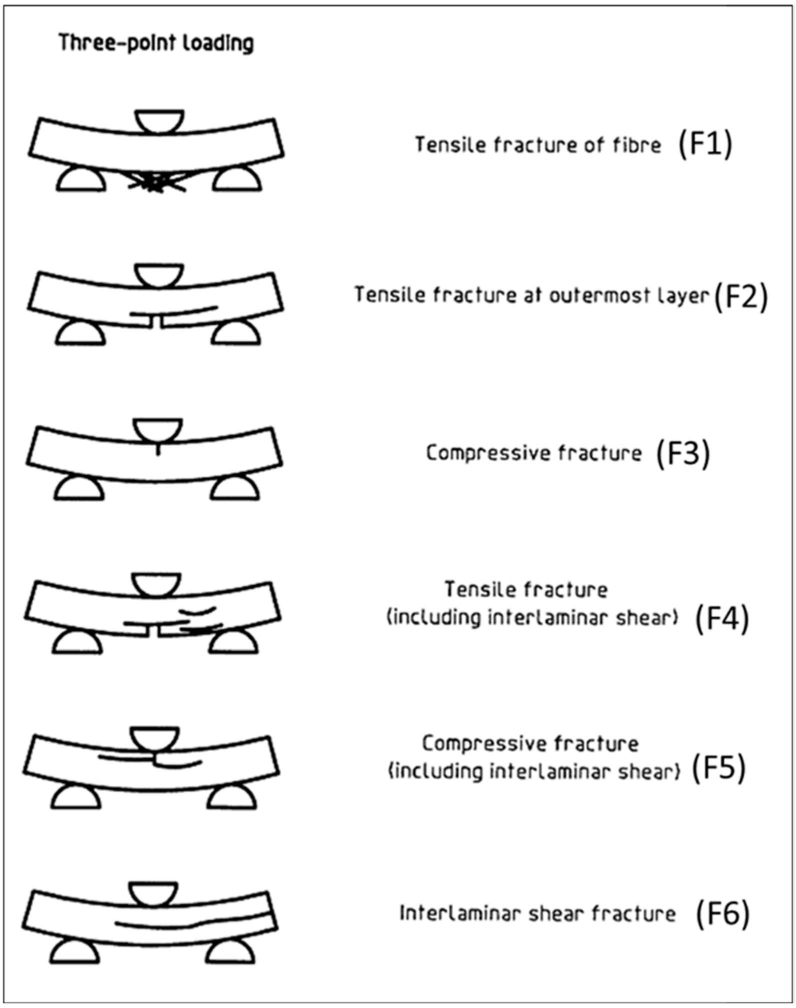

Figure S9. Examples of possible flexural failure modes [1].

References

[1] In: BS EN ISO 14125, Fibre-reinforced plastic composites—Determination of Flexural properties. 1998.
